# Mycoplasma contamination in the 1000 Genomes Project

**DOI:** 10.1186/1756-0381-7-3

**Published:** 2014-04-29

**Authors:** William B Langdon

**Affiliations:** 1 Department of Computer Science, University College London, Gower Street, London WC1E 6BT, UK

**Keywords:** Molecular biology, Microbiology, genetics, metagenomic, Data mining, Next-generation DNA sequencing, Data cleansing, High throughput, Solexa, 454, SOLiD

## Abstract

**Background:**

*In silco* Biology is increasingly important and is often based on public data. While the problem of contamination is well recognised in microbiology labs the corresponding problem of database corruption has received less attention.

**Results:**

Mapping 50 billion next generation DNA sequences from The Thousand Genome Project against published genomes reveals many that match one or more Mycoplasma but are not included in the reference human genome GRCh37.p5. Many of these are of low quality but NCBI BLAST searches confirm some high quality, high entropy sequences match Mycoplasma but no human sequences.

**Conclusions:**

It appears at least 7% of 1000G samples are contaminated.

## Background

Mycoplasma are tiny bacteria which readily grow in cell culture media. They have small genomes. Contamination of molecular biology laboratories by them is widespread [1]. Their small size makes them hard to detect. Depending upon medium, Mycoplasma contamination rates of 1% to 15–35% (or even higher) have been reported [2]. Mycoplasma contamination can render cell line gene expression measurements unreliable [1]. Many labs routinely sterilised their equipment to counter it. About 1% of published NCBI’s Gene Expression Omnibus (GEO) [3] GeneChip data appear to be contaminated [4,5]. Indeed wet lab contamination is so wide spread that Mycoplasma genes have managed to jump the silicon barrier and get themselves incorporated into international data banks as *Human* genes [6].

GEO contains gene expression data, here we start to look for similar contamination in genome studies. The 1000 Genomes Project [7] is an international collaboration which has mapped in whole or in part the genomes or more then 2500 individuals and published studies of SNPs and other human genetic variations. We selected The 1000 Genomes Project, since it investigates human genetic material, is widely respected, it covers many sites with diverse data sources and has made available vast quantities of its raw data.

## Results and discussion

**Figure 1:**
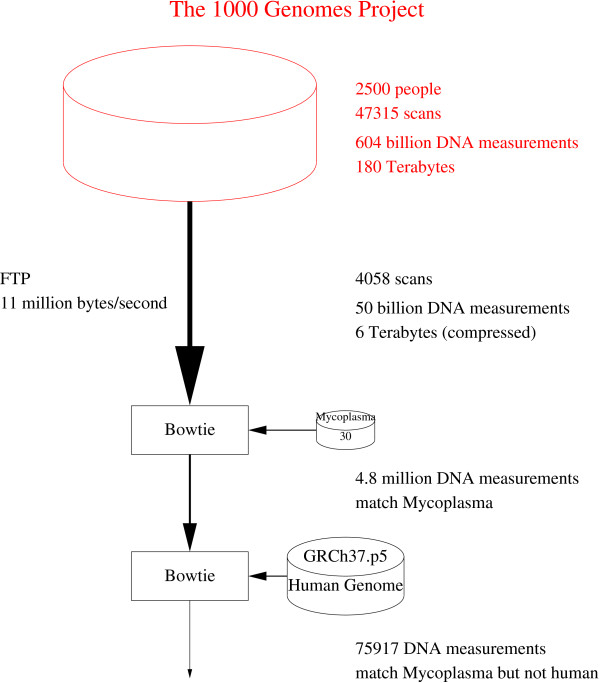
**Schematic showing major data flows in Mycoplasma analysis of The Thousand Genome Project (top color).** A random sample (≈8%) of next generation scan are copied across the Internet to the computer at UCL (black). Bowtie [8] is used to extract individual and paired-end DNA measurements which match one or more of the thirty published Mycoplasma genomes (Additional file 1: Table S1). Bowtie is used a second time to exclude DNA measurements which match the reference human genome, leaving 75 879 Mycoplasma DNA measurements from 2055 scans of the 4058 downloaded.

**Figure 2:**
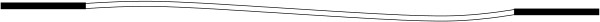
**The 1000 Genomes Project uses a variety of next generation DNA sequencing machines (also known as scanners).** Some use paired-end DNA strands (schematic above). These scans give the DNA base sequence at both ends (shown as solid black). Only the approximate number of bases between the ends is known. The scanner does not report the sequence of bases between the ends. With paired-end scanners, the two ends together are referred to as a single “DNA measurement”. Other scans only contain the sequence of bases at one end of the DNA strand. In these cases there is also one “DNA measurement” per DNA molecule.

Bowtie (version 0.12.7) [8] found 4 803 930 DNA measurements in a random sample which match one or more Mycoplasma genomes (see Figures 1 and 2)^a^. Almost all these also matched somewhere in the reference human genome, leaving 75 879 which match Mycoplasma but do not appear to be human. These are non-uniformly clustered in 51.6% of individual DNA samples.

NextGen scanners are noisy. So, on the assumption that errors are independent, typically multiple (e.g. 3) scans are run. However non-uniform clusters of errors indicate that they are not independent and therefore redundant scans may not resolve the problem. Noise may be part of the reason why Bowtie reports about 30% fail to align to the human genome. However some of these unmatched DNA measurements may not be simply due to noise. These are the ones we investigate to see if they could be due to Mycoplasma contamination.

### Number of mismatches between the 1000 Genomes Project DNA and Mycoplasma

**Figure 3:**
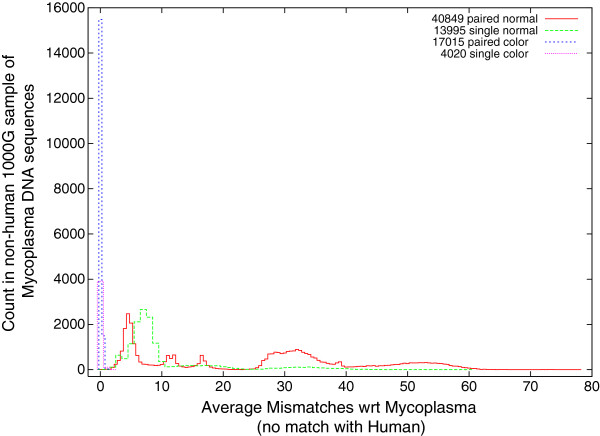
**Distribution of mismatches in matches against Mycoplasma genomes for 75 879 DNA sequences from The 1000 Genomes Project which do not match the reference human genome.** Of these 40 849 are paired end DNA sequences from Illumina or 454 Life Sciences (not colorspace) next generation scanners, 13 995 are single ended also produced by Illumina or 454 Life Sciences scanners, 17 015 paired end produced by Life Technologies SOLiD colorspace scanners and the remaining 4 030 were also reported by Life Technologies SOLiD colorspace but are single ended DNA sequences.

**Figure 4:**
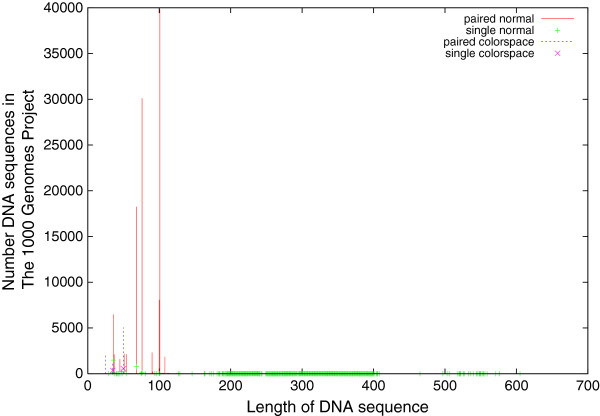
**Lengths of DNA sequences for The 1000 Genomes Project.** Mostly measurements have two paired ends. The mode is for each end to have 101 DNA base pairs. Again, as with Figure 3, data are split by type of sample preparation and sequencing machine.

Figure 3 shows that although Bowtie finds matches within one or more Mycoplasma genomes for 75 879 DNA sequences drawn from The 1000 Genomes Project (but does not match them with the human reference genome) the accuracy of the match varies considerably. Many match a Mycoplasma exactly. These are shown on the left of Figure 3. For others, Bowtie reports up to 78 mismatches. Note the long thin tail to the right in Figure 3. Figure 3 also breaks these data down into pair end and single DNA strands and Solexa coding type (normal v. SOLiD colorspace). Colorspace encoding is described on page 48. Although the colorspace encoding represents a small fraction of the whole data, of the DNA measurements which match Mycoplasma only and for which Bowtie reports (on average) three or fewer mismatches, 93% of them are colorspace encoded. Notice however colorspace sequences tend to be much shorter, see Figure 4. On average, if affected, colorspace scans contain many more affected DNA measurements than normally coded Solexa scans. See columns 3–4 of Table 1. Overall ten percent of The 1000 Genomes Project scans contain sequences which match Mycoplasma well (i.e. on average ≤ 3 mismatches) but do not appear in the reference human genome, last figure in Table 1.

**Table 1:** Approximately 8% of The 1000 Genomes Project was selected at random and downloaded

	**Type**	**Mycoplasma**	**Affected**	**Scans**	**Fraction**
		**sequences**	**scans**	**downloaded**	**of scans**
Pair	Ordinary	797	106	3454	3%
Pair	Colorspace	17 015	111	145	77%
Single	Ordinary	752	108	384	28%
Single	Colorspace	4 020	72	75	96%
	Totals	22 584	397	4058	10%

Of the 53 billion DNA measurements down loaded, 22584 (bottom column 3) match Mycoplasma (with on average three or fewer mismatches) but do not match at all the reference human genome GRCh37.p5. Typically samples infected with Mycoplasma are immediately destroyed. Column 4 gives the number of scans with at least one affected DNA measurement. The final column gives this as a percentage of scans of the same type.

### Quality of The 1000 Genomes Project DNA measurements

Solexa data, like that from other nextGen scanners, are inherently noisy. Solexa provides an estimate of the signal to noise ratio (expressed as log10) per base position in each DNA sequence. (For example, a quality of 0.5 (*S*/*N*=3.16) means the returned base is more likely than the other 3 combined)^b^. This can easily mount up to several hundred quality values. To stably condense these into a manageable statistic, we ignore the worse and second to worst base in each DNA sequence and use the third worst. For paired end data, we use worst of the two ends.

**Figure 5:**
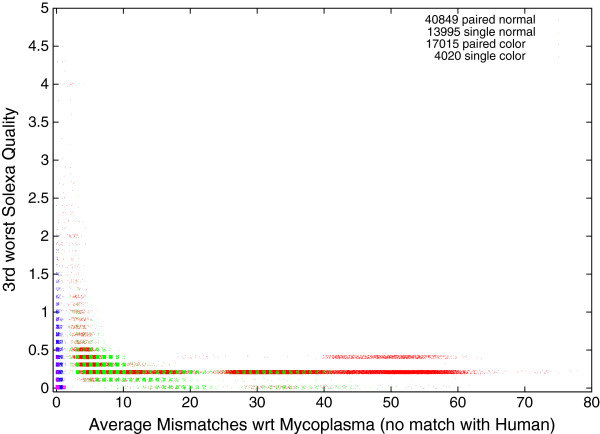
**Quality of 75 879 sequences from The 1000 Genomes Project which match one or more Mycoplasma genomes but do not match the reference human genome.** Horizontal and vertical noise added to spread data. Most sequences which fail to match GRCh37.p5 but do match one or more species of Mycoplasma are of low quality. Nevertheless an important fraction are of high quality and match Msycoplasma with no or few mismatches. As with Figure 3, data are split in four by type of sample preparation and sequencing machine.

**Figure 6:**
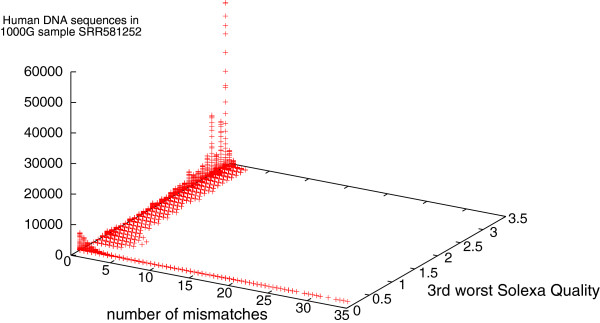
**Quality v. accuracy of match (horizontal) for 1 762 302 DNA sequence pairs which match the human reference genome.** (From an example 1000 Genomes Project paired-end run). Showing typically large numbers (>5) of mismatches are only reported for poor quality data.

If we compare the quality of DNA measurements which match Mycoplasma but which do not occur in the reference human genome (Figure 5) with those which do match GRCh37.p5 we see in both cases measurements with a large numbers of mismatches only occur in low quality data. Figure 6 reports a typical run. Further Figure 5 makes it plain that most of the DNA measurements which match Mycoplasma but which do not occur in the reference human genome contain at least three poor quality values. Nonetheless in our large sample of more than 50 billion DNA measurements drawn randomly from The 1000 Genomes Project, there are 1944 measurements with a quality above 0.5 (which match one or more Mycoplasma genomes with ≤3 mismatches). They occur in 269 scans, this is 7% of our sample, see last number in Table 2.

**Table 2:** In our random download of The 1000 Genomes Project, 1944 high quality DNA measurements (i.e. no more than three bases with quality worse than 0.5) match Mycoplasma (with on average three or fewer mismatches) but do not match at all the reference human genome (bottom column 3)

	**Type**	**High quality**	**Affected**	**Scans**	**Fraction**
		**Mycoplasma sequences**	**scans**	**downloaded**	**of scans**
Pair	Ordinary	542	87	3454	3%
Pair	Colorspace	1042	63	145	43%
Single	Ordinary	234	78	384	20%
Single	Colorspace	126	41	75	55%
	Totals	1944	269	4058	7%

As with Table 1, column 4 gives the number of scans with at least one affected DNA measurement. The right most column gives the percentage of affected scans by type.

### Entropy of The 1000 Genomes Project DNA matching Mycoplasma

**Figure 7:**
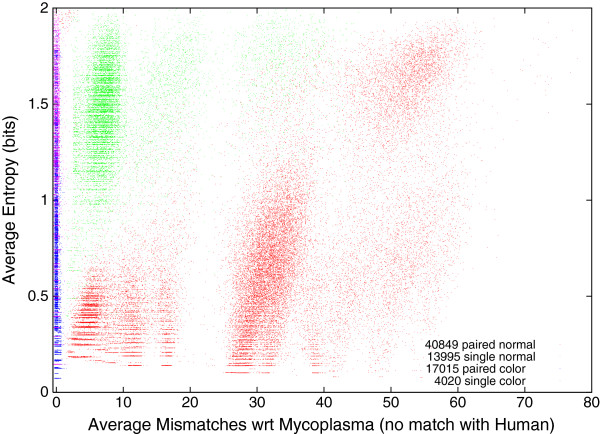
**Entropy per DNA base of 75 879 sequences from The 1000 Genomes Project which match one or more Mycoplasma genomes but do not match the reference human genome.** (See also Figure 9. Horizontal noise added to spread data). As with Figure 3, data are split in four by type of sample preparation and sequencing machine.

**Figure 8:**
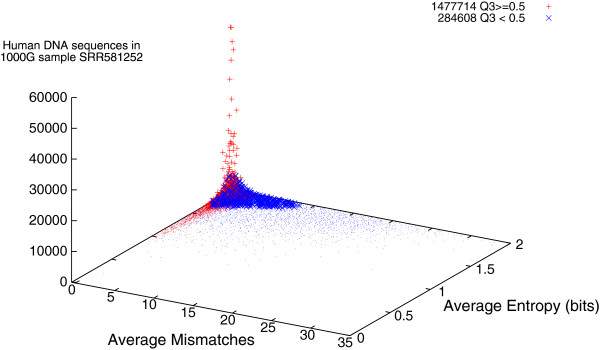
**Entropy v. number of mismatches for 1 762 302 DNA pairs which match the human reference genome.** (From the same example 1000 Genomes Project paired-end run as in Figure 6). Most DNA measurements which match GRCh37.p5 are not repetitive (i.e. have high entropy). Also low quality (×) measurements tend to have more mismatches.

**Figure 9:**
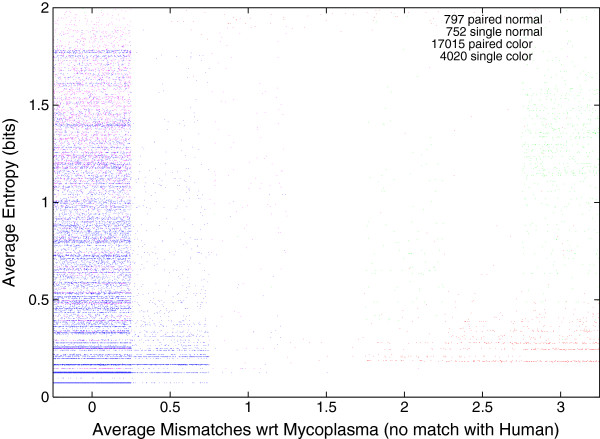
**Entropy per DNA base of 22 584 sequences from The 1000 Genomes Project which match one or more Mycoplasma genomes with 3 or fewer mismatches but do not match the reference human genome.** (Detail of Figure 7. Horizontal noise added to spread data). As with Figure 3, data are split in four by type of sample preparation and sequencing machine.

Figure 7 shows that the exactness with which the DNA measurements match Mycoplasma and the entropy (incompressibility) of its sequences appears to be unrelated. For the very much larger volume of sequences which do match the human reference genome, entropy also plays little role. Instead large numbers of mismatches occur only in low entropy sequences. (Figure 8 plots data from a typical 1000 Genomes Project run). Although Bowtie reports a match, in some cases Bowtie must change many (up to 78) individual DNA bases to get an exact match between the measured DNA sequences and one of the published Mycoplasma genomes. Low entropy (compressible) DNA sequences are highly repetitive. Many real genomes have highly repetitive regions. A highly repetitive simple DNA pattern (even if it exactly matches against a genome) is liable to fall in repetitive region of a (published) genome, where coverage is liable to be patchy [9]. See also Figure 9, which concentrates on Mycoplasma only DNA measurements which match Mycoplasma genomes well.

### Confirming Bowtie with NCBI BLAST

Rather than trying to run BLAST [10] on several thousand DNA strings, we used entropy, a higher quality threshold and exact matching, to choose the best sequences and then ran BLAST on these. In detail, we used a quality threshold above 1.3, we ignored repetitive DNA sequences (i.e. average entropy below 1.0) and requiring at least one exact match against one of our Mycoplasma genomes. This gives seven measurements, none of which is from a SOLiD colour space scanner. See Table 3. BLAST provides strong evidence that these DNA measurements are really from one or more Mycoplasma or similar species.

**Table 3:** High quality, non-repetitive DNA measurements from The 1000 Genomes Project which match one or more published Mycoplasma genomes but which do not match the reference human genome

2.0	2.9	ERR009050.2605525	GCCGTAACTATAACGGTCCTAAGGTAGCGAAATTCCTTGTC E=7 10^−13^	S16 23S ribosomal RNA
2.0	2.3	ERR002459.4464466	ACGGTTTTCAAGACCGTTCCCTTCAGCCAGACTTGG E=5 10^−10^	Transfer RNA-Ser
			CCTGACGGTTTTCAAGACCGTTCCCTTCAGCCAGAC E=5 10^−10^	
1.8	2.1	ERR013159.14600701	CGCTTTCATTGTTCCGCCAGTAGCTAAAACATCATCAATAATTGCTACTTTTTGGCCTTTTTTCAACATATTAGTTT	
			GGATTTCTAGAGTTGATTTACCATATTCTAA E=3 10^−52^	
			TTTTTGGCCTTTTTTCAACATATTAGTTTGGATTTCTAGAGTTGATTTACCATATTCTAAATCATACTCAAAACTAAT	
			AACGTCTCCTGGTAATTTTTTAGGTTTTCT E=3 10^−52^	
1.8	2.0	ERR013159.12593030	GAGCTTGTTTTTCGTATTTTTCAATTTCTATTTCGTCATTGATTTGTCAATTTGGTAAATTTGTGTTTTCGCTATCAGG	
			TTTGGTTAGTTTAAAATAACCATCAAAAG E=2 10^−10^	
			AGGTTTGGTTAGTTTAAAATAACCATCAAAAGTAATTATTGAACCAGAAAGATAAAATTTGTGTTCTTGATTTAAA	
			AATTCATAACGTGTAATTTGTCTTTCAGGAAC E=3 10^−52^	
1.7	2.2	ERR013159.18901091	GGTCAAGTTTACAACAAAATGTTTGCACTTCAAAAAGAACTAGAAGAACTAGAAGAAAATAAAGAAGAAAATA	
			CTTTAATCAAAGAAGTAGTGAACCAAGAAGATATT E=3 10^−6^	
			AAAAGAACTAGAAGAACTAGAAGAAAATAAAGAAGAAAATACTTTAATCAAAGAAGTAGTGAACCAAGAAGA	
			TATTGCAAATATTGTTTCTAAATGAACAAAAATTCC E=3 10^−9^	
2.0	1.9	ERR013159.7037432	TCTAGAGATACTGCCTGGGTAACCAGGAGGAAGGTGGGGACGACGTCAAATCATCATGCCTCTTACGAGTGGG	
			GCAACACACGTGCTACAATGGTCGGTACAAAGAGA E=3 10^−52^	16S ribosomal RNA
			AGTGGGGCAACACACGTGCTACAATGGTCGGTACAAAGAGAAGCAATATGGTGACATGGAGCAAATCTCAAA	
			AAACCGATCTCAGTTCGGATTGAAGTCTGCAACTCG E=3 10^−52^	
1.9	1.9	ERR022473.14544768	TGCTTTTTTACCTCATGGAGTAAGTGGTGCTTTACGTCCAATTGGTTGTTTACCTTCACCACCACCATGTGGGTGA	
			TCATTTGGGTTCATTACAGAACCTCTAACTGT E=3 10^−52^	Ribosomal protein cluster
			GGTGATCATTTGGGTTCATTACAGAACCTCTAACTGTTGGACGAATACCTAAATGACGATTACGTCCTGCTTTTCC	
			AATGTTAACTAGGTTATGTTCTTCATTTCCTA E=3 10^−52^	

Entropy per bit (column 1), 3rd worst quality (column 2), file and sequence id (column 3) of Solexa DNA strands (column 4). E = chance of random match [10]. Column 5 gives an example gene (see discussion on page 12).

## Conclusions

Here we have analysed DNA sequences directly, rather than gene expression. While the techniques are totally different, there is still considerable scope for sample contamination and sequence comparison, Table 2, suggests at least 7% of public data provided by The 1000 Genomes Project may have some Mycoplasma contamination. However the fraction may be higher due to: overlap in DNA sequence space between Human and Mycoplasma genomes and due to excluding low quality data.

Whilst the problem of contamination of nextGen sequences has been considered before, previous studies, e.g. Jun et al. [11] and Cibulskis et al. [12], have looked at contamination by other members of the same species. Indeed there have been several reports of unexpected personal, i.e. human, DNA in The 1000 Genomes Project public data but no reports of non-human contamination. However we downloaded and scanned a random sample of more than 50 billion DNA measurements from their FTP site and found tens of thousands which may have come from Mycoplasma contamination. Since some DNA sequences have been conserved by evolution, it is possible the contamination is from similar species.

### Implications and ork

Once Mycoplasma is suspected, it may be that individual scans can be clean up relatively easily as cross-species contamination is said to be easily detected ([12], page 2601). Indeed a number of commercial Mycoplasma detection tools are based on looking for Mycoplasma genes [2]. However both current microbiology laboratory [2] and Bioinformatics [1] typically take the robust approach of removing (deleting) all potentially infected materials. Indeed when The 1000 Genomes Project withdraws nextGen data, it withdraws complete scans. That is, it simply discards information on about a billion DNA bases each time a scan is withdrawn.

Raw data from The 1000 Genomes Project are publicly available and are being increasingly widely and diversely used. Whilst noisy data may be acceptable for use by their original owners, who are aware of their limitations, there is an increasing risk of contaminated data being (ab)-used outside the laboratories which initial created them. Indeed with staff-turnover there may be risks associated with using what becomes historical data where their provenance becomes more cloudy. Independent numerical studies could be done. The size of our sample suggests (at least for historical data drawn from the same period) they should yield the same results. However, whilst we have established a lower bound for contamination, future studies should be able to calculate it more precisely. For example, by considering redundant scans and clusters it should be possible to isolate the source and perhaps also provide numerical techniques to mitigate the data [12]. Other studies might also look for other effects and thus extract more scientific knowledge from this valuable resource.

Since Mycoplasma are rampant in modern microbiology laboratories [2] it is no surprise to find some in parts of data from The 1000 Genomes Project. We have identified some samples which have a higher than average chance of being contaminated by Mycoplasma. *In silico* studies should be reinforced by checking the source of the data. We urge each member of The 1000 Genomes Project Consortium (as some are apparently doing [12]), particularly those using single ended colorspace scanners (cf. Table 1) to re-check their procedures. Drexler and Uphoff [2] suggest using at least two detection techniques when checking samples for Mycoplasma.

## Methods

The master index file, sequence.index, which describes all the current 1000 Genomes Project data was down loaded [13]. As of 8 February 2013 there were 47,315 scans available (a further 208 had been withdrawn). They comprised: 39 736 paired-end and 4822 single ended DNA sequence scans plus a further 1611 (paired end) and 938 (single ended) scans which used ABI_SOLID colorspace encoding. 4058 were randomly chosen and down loaded. All the DNA measurements are in fastq format, so they include a quality score per DNA base pair. Each scan contains DNA sequences of the same length. Figure 4 shows the distribution of DNA sequence lengths. Almost all colorspace sequences contain 25, 35 or 50 base pairs, whereas lengths 68, 76, 100 and 101 dominate non-colorspace sequences.

On average: each scan contained 13 million DNA sequences (or pairs of sequences). Even compressed, each file is approximately a gigabyte. (Compression reduces download size by a factor of about 3.1) Paired end scans need two such files. The down load speed was variable, typically between 2.5 10^6^ and 36 10^6^ bytes/second, with a mean of 11 million bytes per second. In total 7547 files were down loaded (6.0 terabytes) containing 51 494 393 834 DNA measurements totalling about 7.5 10^12^ base pairs.

**Figure 10:**
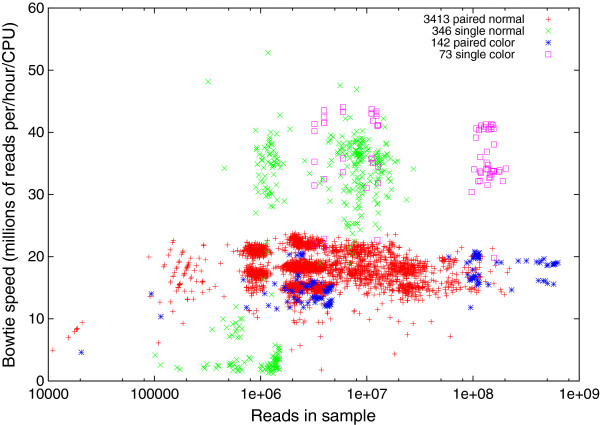
**Speed of Bowtie mapping short nextGen DNA sequences from The 1000 Genomes Project against 30 Mycoplasma genomes (Additional file 1: Table S1).** As expected, Bowtie is typically about twice as fast on single ended (× and □) compared to paired end DNA sequences (+ and *). For the colorspace DNA sequences (* and □) Bowtie uses a colorspace version of its EBWT indexes. Bowtie run on a 32 GB 8 core 3 GHz server. Note log horizontal scale.

We then used Bowtie [8] to find those DNA measurements (i.e. DNA sequences or pairs of DNA sequences) which matched one or more of the published Mycoplasma genomes but do not match the reference human genome GRCh37.p5. See Figure 10. We used all of the Mycoplasma genomes available from NCBI (30 in total. See Additional file 1: Table S1). Apart from using multiple threads -p8, Bowtie’s defaults were used through out. The Bowtie EBWT databases for the normal and colorspace Mycoplasma genomes are both 36 MBytes. Despite including 30 species, due to the small size of Mycoplasma genomes, they are both considerably smaller than that for the two for the human reference genome, which are 2.9 GB for both normal and colorspace. The Bowtie EBWT databases and colorspace databases for the human reference genome GRCh37.p5 include all sequences. I.e., as well as chromosomal DNA, they both include human mitochondrial, “unlocalized” and “unplaced” sequences.

Notice (Figure 10) Bowtie is usually faster on single ended rather than paired double ended DNA sequences (mean 28 v. 18 million sequences per hour per CPU). Although downloading and decompressing the files took 37% of the elapsed time, despite using all 8 CPU cores, almost all the remaining 63% of time was used by Bowtie.

### Estimating entropy

In statistical mechanics, entropy is the degree of disorder in a system [14]. In information theory this translates to the degree or randomness or incompressibility of data, particularly in transmission of messages [15]. entropy=−∑plogp, where *p* is the probability of a sequence of symbols and we sum over all possible symbols. For replicability, the remainder of this section details how we approximate entropy using actual DNA base counts in finite sequences.

In order to have entropy expressed in bits we use log2.

A reasonable estimate of the compressibility of variable length DNA sequences can be made by considering all loss-less coding schemes of up to four bases. The most efficient coding scheme gives the most compressible output. For example, a long sequence of adenine (AAAAAAAAAAAAAAAAAAAA...) can be recoded as a shorter sequence of 00000..., where 0 is one of the new 256 codes needed to represent AAAA–TTTT. Since the coding is loss-less, the encoded sequence contains the same information and so it has the same entropy.

We approximate probability *p* by the actual ratio of each symbol to the number of symbols in the string, *p*=*i*/*l*, so $\text{entropy}=-\sum_{\text{all symbols}}(i/l)\;\log_{2}\;(i/l)$. Where *l* is the length of the encoded string and *i* is the number of each symbol in it. To get the best estimate, we would have to consider all codings. By using the minimum of all 10 possible codings of length up to four DNA bases, we get a reasonable estimate that can deal exactly with not only runs of single bases up to runs of four repeated bases, but gives reasonable estimates with larger repeating sequences. DNA bases which are unknown (i.e. coded as N) are ignored. We use $\text{entropy}=\underset{\text{all codings}}{\min}\left(-\sum (i/l)\;\log_{2}\;(i/l)\right)$. Thus the sequence ACGTACGTACGTACGTACGT, which is highly compressible, has an entropy of −(5/5) log2(5/5)=0. Whereas a simple count of number of bases would show A C G and T each occur 5 times (are present in equal numbers) and so incorrectly would say the string has maximal entropy $-\sum_{i=A,C,G,T}(5/20)\;\log_{2}\;(5/20)=2$. More sophisticated calculations might consider longer potential coding sequences but then the coding tables would be much larger and eventually their information content could no longer be ignored.

### Two base colorspace encoding

Some next generation DNA scanners use a technology which instead of reading DNA sequences one base at a time they use multiple fluorescent dyes to read adjacent (overlapping) pairs of bases. Reduced noise is claimed, since as the pairs overlap, each base is read twice. Data are presented as the initial base followed by transitions from one base type to the next in the sequence (hence needing 4 colours). A potential downside is if an error does occur, the rest of the sequence will be nonsense. Whereas in direct encoding only the erroneous base is effected. It is possible to convert between the two encodings. However because of the different noise characteristics it is usually recommended, as we did, to use tools like Bowtie which can deal with colorspace encoded data directly.

### Selecting a high quality sample to confirm with NCBI BLAST

We used NCBI’s Blast [10] program to confirm our Bowtie results. (We used the default parameters provided by the EBI web interface except we request the first 1000 matches, rather than the first 50 matches). Using BLAST on each of the sequences in Table 3 shows each of the seven high quality DNA measurements (see page 39) do, as expected, match one or more species of Mycoplasma and none matches the reference human genome. In a few cases the second pair matches “Homo sapiens clones”, rather than the human reference sequence. Often these are draft sequences and only in one case (ERR013159.14600701) do both ends of DNA pair match the clone. The final column of Table 3 reports an example of one of the Mycoplasma genes which BLAST finds which match the DNA sequence. In the case of paired end DNA measurements, BLAST has been run separately on both end. The reported gene is matched by both ends. (In three cases an example gene has not been chosen because BLAST matches the whole of, a number of, Mycoplasma genomes). Noting the example gene’s similarity, it is tempting to ascribe some biological meaning to the gene, however BLAST effectively searches all the published DNA sequences and so the similarity may well simply reflect a bias in the published sequences. Ribosomal DNA is highly conserved and has been heavily studied as a tree of life phylogenetic marker of evolutionary inheritance, which makes it one of the more frequent genes in today’s DNA sequence databanks.

We take BLAST’s matches and the lack of BLAST matches against the official human reference genome as confirming our Bowtie results. That is, Table 3 suggests samples ERR009050, ERR002459, ERR013159 and ERR022473 appear to have been contaminated with Mycoplasma. However, of these four, only in one (ERR009050) are there more than a few score DNA measurements which Bowtie matches against Mycoplasma.

## Endnotes

^a^ Some scanners report DNA sequences for both ends of a fragment of DNA. Nonetheless the pair of sequences is considered one “DNA measurement”. See also Figure 2.

^b^ Whilst details depend on the individual manufacturer, essentially each base is allocated a different colour. The brightest colour indicates the base and the quality is estimated from how strong it is compared to the other three colours.

## Competing interests

The author declare that he has no competing interests.

## Supplementary Material

Additional file 1 Mycoplasma Genomes Used. Click here for file
