# Supplementary material for: Mycoplasma contamination in the 1000 Genomes Project
Source: BioData Min. 2014 Apr 29;7:3. doi: 10.1186/1756-0381-7-3 (PMC4022254; doi:10.1186/1756-0381-7-3)
Supplement: Additional file 1 — Mycoplasma Genomes Used. [file 1756-0381-7-3-S1.pdf]

Additional File 1
Mycoplasma Genomes Used

All the Mycoplasma genomes on FTP site `ftp.ncbi.nih.gov` files `genomes/Bacteria/Mycoplasma_*` were down loaded from (30 files, 24 November 2011) and incorporated into a Bowtie EBWT database and a colorspace database.

Table S1: Thirty species of Mycoplasma whose Genomes were used

Genome fasta description	Mycoplasma Complete Genome
gi|148377268|ref|NC_009497.1|	agalactiae PG2
gi|291319937|ref|NC_013948.1|	agalactiae chromosome
gi|193082772|ref|NC_011025.1|	arthritidis 158L3-1
gi|339320528|ref|NC_015725.1|	bovis Hubei-1 chromosome
gi|313678134|ref|NC_014760.1|	bovis PG45 chromosome
gi|83319253|ref|NC_007633.1|	capricolum subsp. capricolum ATCC 27343
gi|240047135|ref|NC_012806.1|	conjunctivae HRC/581 chromosome
gi|294155300|ref|NC_014014.1|	crocodyli MP145 chromosome
gi|308189587|ref|NC_014552.1|	fermentans JER chromosome
gi|319776738|ref|NC_014921.1|	fermentans M64 chromosome
gi|294660180|ref|NC_004829.2|	gallisepticum str. R(low) chromosome
gi|108885074|ref|NC_000908.2|	genitalium G37
gi|321309518|ref|NC_014970.1|	haemofelis str. Langford 1
gi|269114774|ref|NC_013511.1|	hominis ATCC 23114 chromosome
gi|54019969|ref|NC_006360.1|	hyopneumoniae 232
gi|72080342|ref|NC_007332.1|	hyopneumoniae 7448 chromosome
gi|71893359|ref|NC_007295.1|	hyopneumoniae J chromosome
gi|304372805|ref|NC_014448.1|	hyorhinis HUB-1 chromosome
gi|313664890|ref|NC_014751.1|	leachii PG50 chromosome
gi|47458835|ref|NC_006908.1|	mobile 163K
gi|330370665|ref|NC_015407.1|	mycoides subsp. capri LC str. 95010 plasmid pMmc-95010, complete sequence
gi|331703020|ref|NC_015431.1|	mycoides subsp. capri LC str. 95010
gi|127763381|ref|NC_005364.2|	mycoides subsp. mycoides SC str. PG1 chromosome
gi|26553452|ref|NC_004432.1|	penetrans HF-2
gi|13507739|ref|NC_000912.1|	pneumoniae M129
gi|15828471|ref|NC_002771.1|	pulmonis UAB CTIP
gi|344204770|ref|NC_015946.1|	putrefaciens KS1 chromosome
gi|325972867|ref|NC_015155.1|	suis str. Illinois chromosome
gi|325989358|ref|NC_015153.1|	suis KI3806
gi|71894025|ref|NC_007294.1|	synoviae 53
